# Early introduction of tolvaptan after cardiac surgery: a renal sparing strategy in the light of the renal resistive index measured by ultrasound

**DOI:** 10.1186/s13019-015-0372-0

**Published:** 2015-11-02

**Authors:** Tomoko S. Kato, Shunya Ono, Kan Kajimoto, Kenji Kuwaki, Taira Yamamoto, Atsushi Amano

**Affiliations:** Department of Cardiovascular Surgery, Juntendo University School of Medicine, 2-1-1, Hongo, Bunkyo-ku, Tokyo 113-8421 Japan

**Keywords:** Diuretics, Cardiac surgery, Renal function, Tolvaptan, Renal resistive index

## Abstract

**Background:**

Renal failure is a serious complication after cardiac surgery, which can be caused by long-term intravenous (IV) loop diuretic use. Tolvaptan is an oral selective vasopressin-2 receptor antagonist used in patients irresponsive to loop diuretics. We investigated their renal perfusion changes using the resistive index (RI) postoperatively.

**Methods:**

Serial renal RI, echocardiography, and laboratory examinations from 14 patients requiring continuous postoperative IV loop diuretics were reviewed. Eight patients received tolvaptan (Group T) and six received oral loop diuretics before the discontinuation of IV loop diuretics (Group L). The 1^st^ data were obtained between postoperative day 0 and 2, the 2^nd^ when patients were still under IV loop diuretic treatment, the 3^rd^ after the initiation of tolvaptan or oral loop diuretic, and the 4^th^ after the discontinuation of IV diuretics.

**Results:**

The 2^nd^ RI value was higher in Group T than Group L (0.77 ± 0.09 vs. 0.69 ± 0.01, *p* = 0.049) but significantly decreased after tolvaptan administration [0.77 ± 0.09 to 0.65 ± 0.05 (2^nd^ to 3^rd^), to 0.62 ± 0.04 (to 4^th^), both *p* = 0.006], while no such changes were seen in Group L. The serum sodium and albumin levels, and echo-derived tricuspid annular plane systolic excursion increased only in Group T (134.1 ± 1.5 to 138.8 ± 3.2 mEq/L, 3.3 ± 0.3 to 3.7 ± 0.5 g/dL, 16.4 ± 3.6 to 19.7 ± 4.2 mm, all *p* <0.05). The duration of IV loop diuretics tended to be shorter in Group T than Group L (5.6 ± 1.6 vs. 8.7 ± 3.6 days, *p* = 0.051).

**Conclusions:**

Administration of tolvaptan in patients undergoing cardiac surgery may improve their renal perfusion, as reflected by the renal RI measured using renal Doppler ultrasound.

## Background

Renal functional deterioration provokes a serious complication following cardiac surgery that is associated with an increase in morbidity and mortality [1–3]. Although the intravenous (IV) administration of loop diuretics is frequently used during and immediately after cardiac surgery, its high dose requirement is considered a risk factor for worsening renal function as well as worsening heart failure symptoms [4, 5]. The utilization of loop diuretics decreases the glomerular filtration rate and renal blood flow, which is closely linked to the activation of renin-angiotensin-aldosterone systems (RAAS), resulting in the deterioration of cardiac function, especially in patients with heart failure [4–6]. In addition, prolonged exposure to loop diuretics causes renal structural damages to the kidneys, leading to diuretic resistance [4, 7].

Tolvaptan, an oral selective vasopressin two receptor antagonist producing water diuresis, has been recently used in patients with heart failure associated with volume overload refractory to conventional diuretic therapy [8–10]. Since tolvaptan can remove excess water from the body without activating the RAAS or causing serum electrolyte imbalances, co-administration of tolvaptan with loop diuretics is expected to reduce the required dose of loop diuretics and therefore ameliorate adverse events and renal functional deterioration [11, 12]. Shirakabe et al. reported that the immediate administration of tolvaptan in patients with acute decompensated heart failure under the treatment of continuous IV loop diuretics could reduce the amount of loop diuretics and prevent the worsening of acute kidney injury [12].

However, the effect of tolvaptan on preventing renal functional deterioration, especially on intra-renal hemodynamics, by reducing the dose/duration of IV diuretics in patients after cardiac surgery has yet to be elucidated.

The renal resistive index (RI) in the intra-renal arteries measured by Doppler ultrasound is a useful parameter for quantifying the alterations in intrarenal perfusion and renovascular resistance, which is an indicator for the renal functional reserve [13–15]. The advantage of using Doppler ultrasound over B-mode ultrasound includes its ability to detect not only renal morphological abnormalities but also functional ones. B-mode ultrasound can evaluate kidney size, parenchymal thickness, and changes in parenchymal echogenicity, but it is not sensitive enough to diagnose acute renal injury [16]. On the other hand, the measure RI has been reported to be one of the most sensitive parameters detecting disease-derived alterations of renal plasma flow [13–16]. The RI was defined as a ratio of the difference between the maximum and minimum flow velocity to maximum flow velocity of intrarenal arteries [13–16]. A normal RI value in subjects without pre-existing renal disease is reported to be approximately 0.6 [17, 18], and a high RI is known to be a maker of renal functional deterioration [14, 15].

In the present study, we investigated the serial changes in RI and other renal functional parameters in patients after cardiac surgery receiving tolvaptan while being treated with IV loop diuretics, and the results were compared with those conventionally treated with IV loop diuretics followed by a conversion to oral loop diuretics.

## Methods

### Study design

**Fig. 1:**
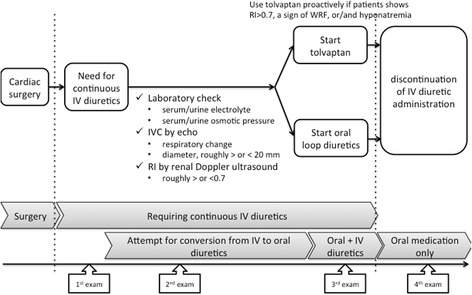
A flow chart of treatment strategies in patients included in the study. *WRF* worsening of renal function, *IV* intravenous, *echo* echocardiography and renal ultrasound

This is a retrospective study at an institution with no definite protocol for using tolvaptan as a part of the renal sparing strategy after cardiac surgery. Still, serial bedside echocardiography together with renal Doppler ultrasound is routinely performed postoperatively, and we aim to decrease the IV diuretic dosage if the patient’s renal RI value is over 0.7. In such cases, oral tolvaptan was initiated to maintain adequate urinary output while the IV diuretic dosage was reduced, if their serum sodium concentration was within a reasonable range and echocardiography did not indicate hypovolemic states (Fig. 1).

As a preliminary analysis, we reviewed data obtained from 14 patients whose RI values were recorded at least four times after cardiac surgery. Because we performed renal Doppler ultrasound only in patients who required continuous intravenous diuretics postoperatively, we were unable to include their preoperative RI values in the present investigation. Among the 14 patients we studied, eight were treated with oral tolvaptan (Group T) and six with oral loop diuretics (Group L) before the discontinuation of IV diuretics. Data including renal Doppler ultrasonography were obtained from each patients at four time points. The 1^st^ data were obtained soon after surgery between postoperative day 0 and 2 (1^st^ exam). The 2^nd^ data were obtained after the 1^st^ exam while patients were still under continuous IV loop diuretic treatment alone (2^nd^ exam), and the subsequent oral diuretic administration including the choice of drugs was considered based on the 2^nd^ exam results. The 3^rd^ data were obtained after the initiation of oral diuretics (tolvaptan or oral loop diuretic) but with concomitant IV loop diuretics (3^rd^ exam). The 4^th^ data were obtained after the patients were successfully weaned from IV loop diuretics and were treated with oral diuretics alone (4^th^ exam) (Fig. 1).

### Ultrasonography

Standard echocardiography and renal ultrasound were performed using the Vivid I digital ultrasound system (GE Medical Systems, Horten, Norway). All measurements obtained were in accordance with recommendations of the American Society of Echocardiography [19, 20]. All echo parameters were averaged for three consecutive beats. For the RI measurements, patients were placed in a supine position, and the flow velocities of intra-renal arteries at the level of corticomedullary junction were measured using pulsed Doppler ultrasonography. By using the highest frequency probe, the sample volume was placed in the lumen of the vessel and the speed–time curve was recorded. The size of the sample volume must be set for interlobar arteries (approximately 1–2 mm) in order to avoid artifacts due to under or over sampling [16]. The RI was calculated as; RI = (peak systolic velocity-end diastolic velocity)/peak systolic velocity [13, 18].

### Statistical analysis

Data are presented as mean ± SD. Normality was evaluated for each variable from normal distribution plots and histograms. Data were compared between the groups using student’s unpaired two-tailed t-test for continuous variables, and the chi-square test for categorical variables. Repeated measures analysis of variance was used with respect to the comparison of parameters for each group of patients obtained at four time points. All data were analyzed using the Statistical Analysis Systems software JMP 11.0 (SAS Institute Inc. Cary, NC, USA).

## Results

### Patients’ characteristics

The clinical characteristics of patients before and at the time of cardiac surgery are summarized in Table 1. Age, gender distribution, and body surface area were not different between the groups. Preoperative laboratory values including the estimated glomerular filtration rate (eGFR) and echo-derived parameters were not significantly different between the groups. The eGFR values in both groups were around 50 mL/min/1.73 m^2^, and one-third of patients in Group L and half in Group T suffered from a chronic kidney disease stage >3b. Preoperative general conditions including heart failure severity, as reflected by NYHA class as well as the EuroScore and Japan Score [21–23] tended to be worse in patients treated with tolvaptan; however, the differences were not statistically different. Patients in Group T less frequently underwent coronary bypass surgery alone than those in Group L. This means patients treated with tolvaptan tended to have a disease affecting their right heart function, although the number of patients in each group was not statistically sufficient.

**Table 1:** Patient characteristics

	Group T (*n* = 8)	Group L (*n* = 6)	*p* value
Age (years)	61.5 ± 13.7	63.5 ± 11.5	0.7813
Men	5 (62.5 %)	3 (50.0 %)	0.1477
Body surface area (m^2^)	1.64 ± 0.11	1.60 ± 0.10	0.5998
NYHA class III or IV (n, %)	4 (50 %)	1 (16.7 %)	0.1977
Preoperative laboratory examinations			
Hb (g/dL)	10.9 ± 1.8	10.4 ± 1.1	0.5397
Na (mEq/L)	139.1 ± 4.0	136.0 ± 3.6	0.1567
Cre (mg/dL)	1.20 ± 0.54	1.02 ± 0.28	0.478
T-Bil (mg/dL)	1.4 ± 0.6	1.02 ± 0.2	0.408
TP (g/dL)	6.2 ± 0.5	6.3 ± 0.2	0.792
Alb (g/dL)	3.5 ± 0.3	3.5 ± 0.2	0.831
eGFR (ml/min./1.73 m^2^)	47.6 ± 26.6	52.9 ± 14.4	0.668
CKD stage 3b or worse (n, %)	4 (50 %)	2 (33.3 %)	0.533
Preoperative echocardiography			
LVEDD (mm)	56.8 ± 19.8	50.3 ± 9.4	0.371
LVEF (%)	43.1 ± 14.0	52.0 ± 6.7	0.180
TAPSE (mm)	16.0 ± 5.0	20.3 ± 3.1	0.086
RVFAC (%)	33.5 ± 7.1	38.3 ± 6.5	0.214
Euroscore	12.6 ± 8.1	8.0 ± 4.0	0.2486
Japan Score	15.9 ± 11.8	10.1 ± 5.1	0.2820
Type of surgery (*p value* based on CABG alone vs. others)			0.0499
CABG alone	0 (0 %)	2 (33.3 %)	
Valvular surgery alone	5 (62.5 %)	3 (50.0 %)	
CABG + Valvular surgery	1 (12.5 %)	1 (16.7 %)	
Pericardiectomy	1 (12.5 %)	0 (16.7 %)	
Pulmonary thrombectomy	1 (12.5 %)	0 (16.7 %)	

Abbreviations not defined in the text; *NYHA* New York Heart Association, *Hb* hemoglobin, *Na* sodium; *Cre* creatinine, *T-Bil* total bilirubin, *TP* total protein, *Alb* albumin, *eGFR* estimated glomerular filtration rate, *CKD* chronic kidney disease, *LVEDD* left ventricular end-diastolic dimension, *LVEF* left ventricular ejection fraction, *TAPSE* tricuspid annual plane systolic excursion, *RVFAC* right ventricular fractional area change, *CABG* coronary artery bypass grafting

### Serial changes in renal RI, echocardiographic and laboratory values

**Fig. 2:**
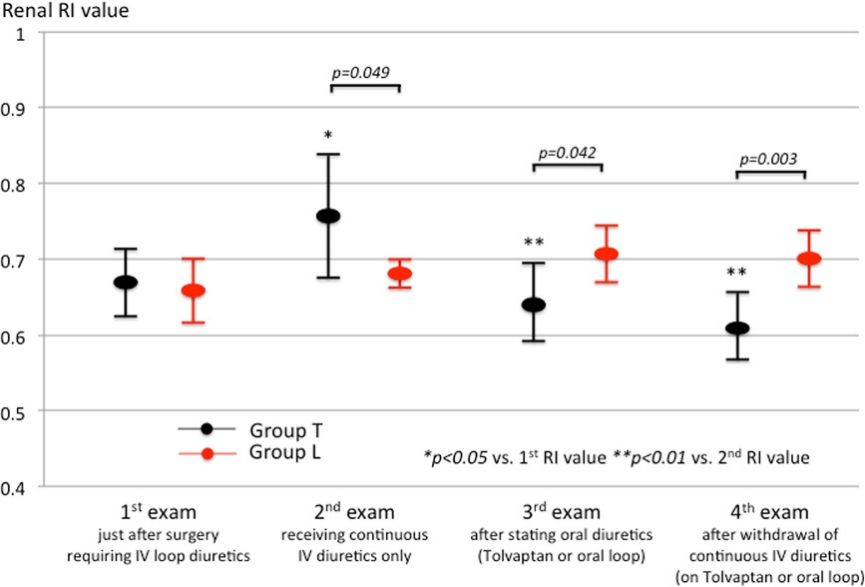
Serial changes in renal RI values in both groups. *Black dots* and *bars* indicate the mean ± SD of RI values in patients receiving tolvaptan (Group T). *Red dots* and *bars* indicate the mean ± SD of RI values in patients receiving oral loop diuretics (Group L)

Serial changes of ultrasound-derived parameters and laboratory values obtained from both groups are shown in Table 2. In Group T, the 2^nd^ RI values increased during continuous IV loop diuretics from the 1^st^ RI *(p = 0.025)*; however, the 3^rd^ and 4^th^ RI values decreased after tolvaptan administration (*p = 0.006, p = 0.006*, vs. 2^nd^ value, respectively) (Fig. 2). In contrast, the serial changes in RI values were not significant in Group L. When we compared the RI values at each time point between the groups, the 2^nd^ RI, which was the value measured during continuous IV diuretic treatment before oral diuretic administration, were higher in Group T than that in Group L. However, after the initiation of oral diuretics (tolvaptan for Group T, loop diuretics for Group L), the RI values in Group L became rather higher than those in Group T (Fig. 2).

**Table 2:** Serial changes in RI values and other examinations

	Group T (*n* = 8)					Group L (*n* = 6)					*p value intergroup comparison*			
	1^st^ exam	2^nd^ exam	3^rd^ exam	4^th^ exam	*ANOVA p value*	1^st^ exam	2^nd^ exam	3^rd^ exam	4^th^ exam	*ANOVA p value*	1^st^ exam	2^nd^ exam	3^rd^ exam	4^th^ exam
Renal ultrasound														
Renal RI	0.67 ± 0.05	0.77 ± 0.09	0.65 ± 0.05	0.62 ± 0.04	*0.0002*	0.66 ± 0.04	0.69 ± 0.01	0.71 ± 0.05	0.70 ± 0.04	*0.2008*	*0.816*	***0.049***	***0.043***	***0.002***
*p value;* vs. *1*^*st*^ *exam*	–	***0.025***	*0.425*	*0.093*		–	*0.203*	*0.096*	*0.196*					
*p value;* vs. *2*^*nd*^ *exam*	–	***–***	***0.006***	***0.006***		–	**–**	*0.341*	*0.485*					
Echocardiography														
LVEDD (mm)	58.5 ± 19.8	55.1 ± 15.4	57.2 ± 16.5	56.1 ± 17.6	*0.9823*	50.3 ± 9.4	51.3 ± 10.3	51.0 ± 11.7	46.5 ± 6.7	*0.8112*	*0.371*	*0.614*	0.446	*0.230*
LVEF (%)	45.1 ± 15.2	44.9 ± 14.5	45.0 ± 14.0	47.0 ± 15.3	*0.9902*	53.3 ± 8.3	52.8 ± 9.5	55.8 ± 11.7	52.0 ± 0.3	*0.9150*	*0.249*	*0.266*	*0.151*	*0.494*
IVC diameter (mm)	23.1 ± 5.4	23.5 ± 3.2	21.5 ± 3.6	19.8 ± 3.2	*0.2336*	23.1 ± 5.4	21.5 ± 2.7	21.2 ± 3.2	18.2 ± 2.6	*0.1475*	*0.379*	*0.240*	*0.860*	*0.439*
*p value;* vs. *1*^*st*^ *exam*	–	*0.798*	*0.318*	***0.021***		–	*0.541*	*0.793*	***0.023***					
*p value;* vs. *2*^*nd*^ *exam*	–	–	***0.049***	***0.004***		–	–	*0.783*	***0.036***					
TAPSE (mm)	16.4 ± 3.6	17.5 ± 3.4	19.0 ± 4.1	19.7 ± 4.2	0.3165	19.8 ± 4.4	21.8 ± 2.8	21.5 ± 3.4	21.5 ± 3.8	*0.7759*	*0.129*	***0.026***	*0.252*	*0.439*
*p value;* vs. *1*^*st*^ *exam*	–	*0.380*	***0.041***	***0.015***		–	*0.216*	*0.387*	*0.419*					
*p value;* vs. *2*^*nd*^ *exam*	–	–	*0.127*	*0.087*		–	–	*0.771*	*0.813*					
Laboratory examinations														
Hb (g/dL)	10.0 ± 1.4	9.7 ± 1.4	10.0 ± 1.1	10.5 ± 1.5	*0.6340*	9.3 ± 0.6	9.1 ± 0.7	9.9 ± 1.2	10.4 ± 1.1	*0.0893*	*0.259*	*0.369*	*0.920*	*0.881*
*p value;* vs. *1*^*st*^ *exam*	–	*0.456*	*0.977*	*0.323*		–	*0.567*	*0.267*	*0.130*					
*p value;* vs. *2*^*nd*^ *exam*	–	–	*0.383*	*0.052*		–	–	*0.283*	*0.110*					
Na (mEq/L)	136.3 ± 3.9	134.1 ± 1.5	136.4 ± 4.1	138.8 ± 3.2	*0.0745*	135.3 ± 2.2	135.0 ± 3.9	134.7 ± 2.3	134.7 ± 2.2	*0.9699*	*0.616*	*0.571*	*0.384*	***0.019***
*p value;* vs. *1*^*st*^ *exam*	–	*0.093*	*0.941*	*0.112*		–	*0.876*	*0.543*	*0.328*					
*p value;* vs. *2*^*nd*^ *exam*	–	–	*0.170*	***0.005***		–	–	*0.797*	*0.830*					
Cre (mg/dL)	1.1 ± 0.6	1.3 ± 0.7	1.2 ± 0.5	1.1 ± 0.4	*0.8967*	1.2 ± 0.5	1.2 ± 0.5	1.3 ± 0.3	1.3 ± 0.3	*0.9180*	*0.841*	*0.914*	*0.584*	*0.271*
*p value;* vs. *1*^*st*^ *exam*	–	*0.079*	*0.190*	*0.502*		–	*0.667*	*0.224*	*0.309*					
*p value;* vs. *2*^*nd*^ *exam*	–	–	*0.388*	*0.189*		–	–	*0.553*	*0.572*					
TP (g/dL)	6.0 ± 0.6	6.0 ± 0.5	6.0 ± 0.5	6.3 ± 0.4	*0.9909*	6.1 ± 0.4	6.0 ± 0.4	6.0 ± 0.5	6.1 ± 0.3	*0.9763*	*0.923*	*0.880*	*0.987*	*0.308*
*p value;* vs. *1*^*st*^ *exam*	–	*0.649*	*0.705*	*0.116*		–	*0.363*	*0.259*	*0.765*					
*p value;* vs. *2*^*nd*^ *exam*	–	–	*0.756*	***0.042***		–	–	*0.862*	*0.275*					
Alb (g/dL)	3.5 ± 0.4	3.3 ± 0.3	3.6 ± 0.2	3.7 ± 0.5	*0.2227*	3.4 ± 0.2	3.2 ± 0.3	3.4 ± 0.2	3.5 ± 0.1	*0.0987*	*0.681*	*0.660*	*0.146*	*0.380*
*p value;* vs. *1*^*st*^ *exam*	–	*0.174*	*0.329*	*0.351*		–	*0.056*	*0.998*	*0.555*					
*p value;* vs. *2*^*nd*^ *exam*	–	–	***0.026***	*0.059*		–	–	*0.071*	*0.073*					
Duration requiring IV loop diuretics (days)	5.6 ± 1.6					8.7 ± 3.6					0.051			

Abbreviations not defined in the text; *LVEDD* left ventricular end diastolic diameter, *LVEF* left ventricular ejection fraction, *IVC* inferior vena cava, *TAPSE* tricuspid annular plane systolic excursion, *Hb* hemoglobin, *Na* sodium, *K* potassium, *Crea* creatinine, *TP* total protein, *Alb* albumin; Bold pvalues are significant at the 0.05 level.

**Fig. 3:**
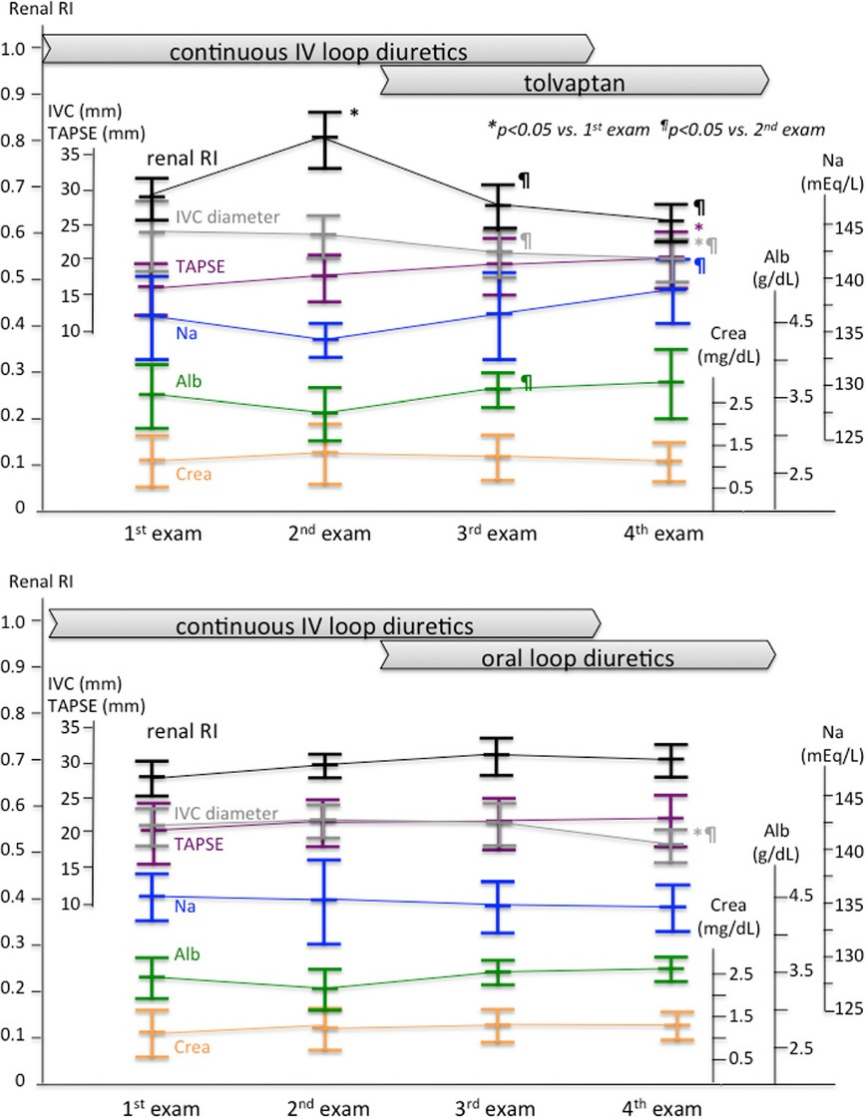
Serial changes in ultrasound and laboratory parameters obtained from patients receiving tolvaptan (*upper*) and those obtained from patients receiving oral loop diuretics (*lower*). *Black dots* and *lines* indicate the RI values, *gray dots* and *lines* indicate IVC diameters, *purple dots* and *lines* indicate TAPSE, *blue dots* and *lines* indicate serum sodium concentrations, *green dots* and *lines* indicate serum albumin levels, and *orange dots* and *lines* indicate serum creatinine levels. *Na* sodium, *Alb* albumin, *Crea* creatinine

The left ventricular end-diastolic dimension (LVEDD) and left ventricular ejection fraction (LVEF) did not change throughout the serial evaluation either in Group T or Group L. In both groups, the maximum diameter of the inferior vena cava (IVC) decreased at the 4^th^ exam compared to the initial and 2^nd^ measurements (Fig. 3). The LVEDD, LVEF, and IVC diameter at each time point were not significantly different between the groups. The tricuspid annular plane systolic excursion (TAPSE) increased throughout the evaluation only in Group T [Fig. 3]. The comparison of TAPSE between the groups showed that the initial measurement of TAPSE tended to be lower in Group T, and the 2^nd^ measurement was statistically lower in Group T than Group L.

Laboratory examination revealed that hemoglobin concentrations tended to increase through the serial measurements in both groups, but the changes were not statistically significant. The serum sodium concentration increased only in Group T. The sodium levels at 4^th^ measurements were higher than the 2^nd^ measurements in Group T, but such changes were not seen in Group L (Fig. 3). The comparison of sodium concentrations at each time point between the groups showed that the values did not differ at 1^st^, 2^nd^, or 3^rd^ measurements, but it was higher in Group T than Group L at the 4^th^ measurements. Neither of the groups showed changes in creatinine levels, and the values at each time point were not significantly different between the groups. Both total protein and albumin concentrations increased at the 4^th^ measurements compared to the values at the 2^nd^ measurements in Group T, but such changes were not seen in Group L (Fig. 3).

The duration of continuous IV diuretics treatment tended to be shorter in Group T than Group L, but the difference was not statistically significant.

## Discussion

In this preliminary investigation, we focused on the serial changes of renal RI values in patients requiring continuous IV diuretics post-cardiac surgery, who were treated with the co-administration of tolvaptan for early discontinuation of IV diuretics and renal functional preservation. Based on the fact that a high RI is reported to be indicative of a decrease in renal perfusion which would elicit renal functional deterioration [13–15, 17], we considered starting tolvaptan but not oral loop diuretics in patients who showed RI elevations and/or those with fluid overload associated with hyponatremia.

We have shown that, (i) the renal RI values decreased in patients treated with tolvaptan administration, while the values did not change in patients treated with oral loop diuretics; (ii) not only serum sodium concentrations but total protein and albumin levels also increased after tolvaptan administration, suggesting that tolvaptan effectively reduces “excessive” body fluid; (iii) tolvaptan would be useful for shortening the duration of continuous IV diuretics requirement after cardiac surgery, although we failed to prove it statistically due to an insufficient number of studied patients.

Renal dysfunction is a serious complication following cardiac surgery, which has a strong impact on short- and long-term morbidity and mortality [1–3]. Cardiopulmonary bypass (CPB) usage during cardiac surgery is associated with an increased risk of acute renal dysfunction [24, 25]. This is due to a decrease in renal blood flow caused by hypotension under extracorporeal circulation and low cardiac output immediately after weaning from CPB, in addition to the inflammatory cytokines and ischemic reperfusion injury [3, 24, 25]. We recently reviewed over 1500 patients undergoing cardiac surgery at our institution and found that CPB was a strong risk factor for the postoperative requirement of renal replacement therapy [26]. Therefore, we believe that keeping sufficient renal perfusion after cardiac surgery is important in protecting renal function, especially from the possible damage caused by CPB. Indeed, all patients whom we treated with tolvaptan in order to avoid further deterioration of renal function caused by prolonged IV loop diuretics in the present study underwent cardiac surgery using CPB.

The renal RI is defined as a ratio of the difference between the maximum and minimum (end-diastolic) flow velocity to maximum flow velocity measured by renal Doppler ultrasound [13, 18]. Because this is the ratio of flow velocity, the results are independent from the types of ultrasound machines. We routinely perform serial bedside echocardiography postoperatively to evaluate hemodynamics and to adjust the necessary medications; therefore, adding renal Doppler ultrasounds to the routine echo was simple and not at all time consuming. The RI is a well-known indicator of renal perfusion, which is useful for the prediction of renal function [13–15, 17, 27, 28]. In the present observation, the RI values increased during continuous IV diuretic infusion, which reflected the deterioration of renal perfusion, and normalized following tolvaptan administration. In contrast, patients who were treated with IV and oral loop diuretics did not show a decrease in RI. This may indicate a possible renal sparing effect of using tolvaptan to reduce the duration/dosage of loop diuretics. We failed to show the difference between serum creatinine levels of the two groups after discontinuation of IV loop diuretics; however, we assume that tolvaptan would be beneficial for maintaining the renal functional reserve, as reflected by a decrease in the RI observed in group T. Indeed, the volume overload status was successfully corrected in both groups according to the significant decrease in IVC diameter during the study period in both groups. Even though we admit that the number of cases was too small and the patients’ backgrounds were not homogeneous between the groups, tolvaptan treatment may have the potential to correct fluid overload without affecting renal perfusion. This result may be associated with the shortening of the duration as well as the reduction of cumulative doses in IV loop diuretic requirements [12]. Furthermore, the tolvaptan administration successfully corrected hyponatremia and hypoalbuminemia, both of which are known to be associated with poor prognosis in heart failure patients [29, 30], although we have not yet investigated the long-term effect of tolvaptan usage in this preliminary analysis.

The timing of tolvaptan initiation is a difficult issue. Loop diuretics are the most commonly-used diuretics to treat volume overload, and are almost always administered intravenously for patients in the intensive care unit (ICU) including post-cardiac surgery. A multicenter, multinational, observational study reported that about 70 % of patients in the ICU receive diuretics, with furosemide being the choice of 98 % [31]. However, the dose of loop diuretics leads to a decrease in renal blood flow and is an independent negative predictor of outcome in patients with heart failure [32]. Loop diuretic-induced hyponatremia also provokes poor prognosis [5, 33]. Unlike loop diuretics, tolvaptan does not activate the renin-angiotensin-aldosterone (RAAS) system and augments water excursion without changes in renal hemodynamics. Therefore, tolvaptan usage combined with reduced dosage of loop diuretics can be a reasonable renal sparing strategy. Shirakabe et al. recently reported that the immediate administration of tolvaptan can prevent the progression of renal dysfunction and improve survival in patients with acute decompensated heart failure [12]. In their study, patients who received tolvaptan immediately after admission together with intravenous loop diuretics ended up requiring smaller amounts of furosemide than those treated conventionally. The ACTIVE-HF trial revealed that tolvaptan treatment reduce mortality when it was initiated within 48 h [34], whereas the EVEREST trial failed to show the survival benefit of tolvaptan treatment when it was administrated more than 48 h after the hospitalization [35]. This may indicate that the beneficial effect of tolvaptan on renal functional preservation as well as the prognosis is more expected when it is administrated in the early phase of fluid management, rather than when it is used after sufficient conventional diuretic therapy. Therefore, we may be able to suggest that tolvaptan treatment, as a renal sparing strategy, should be initiated without waiting for the development of signs of worsening renal failure such as a slight increase in creatinine levels. In other words, creatinine-guided or diuretic dosage-guided tolvaptan initiation may not be always appropriate. In our study, we utilized renal Doppler ultrasound-derived RI values as a guide to decide the timing of tolvaptan initiation. The RI values can be used to discriminate functional/reversible acute kidney injury (AKI) from organic/persistent AKI. The functional AKI is characterized by a reduction in renal perfusion and is rapidly reversible if promptly treated [16]. Indeed, as we applied RI values in the present study, previous studies also reported that RI is a good predictor of the onset of AKI in the early postoperative period of patients undergoing cardiac surgery [36, 37] Considering the high sensitivity and specificity (both >80 %) of the RI as an indicator for persistent AKI [16, 38], as well as the fact that it does not require blood or urine samples and can be performed at the bedside, it could be used to determine the optimal timing of initiating tolvaptan to reduce loop diuretics.

The present study had several limitations. First, this is a single-center, retrospective observational analysis based on a small number of patients. Because this is a preliminary report to investigate the utility of serial RI measurements, we failed to include preoperative RI measurements in the present cohort. However, considering the fact that the RI is a useful parameter to predict not only the worsening but also the recovery of renal function by reflecting the renal functional reserve [13–15], we are now planning to include renal Doppler ultrasound as a part of the preoperative evaluation in patients undergoing cardiac surgery at our institution in a routine manner. In addition, operative profiles were not homogeneous among the groups. However, we believe that our present report may reflect the discretion in the real clinical world about diuretic usage and the attempt of sparing renal function. We cannot emphasize that patients receiving tolvaptan in the present study tended to have fluid accumulation associated with right-sided heart failure; however, we assume that tolvaptan may be a potent therapy in patients with an upregulation of vasopressin derived from persistent renal congestion. Second, the exact timing of obtaining the data was different between the groups, although the conditions of patients under either of the targeted drug usages were consistent. Third, we did not include information on drug usages other than diuretics such as inotropic agents and beta-blockers. Finally, we reviewed only the short-term clinical course and did not review long-term outcomes.

## Conclusions

In conclusion, the administration of tolvaptan after cardiac surgery in patients who require continuous IV diuretics may improve their renal perfusion, as reflected by the renal RI measured using renal Doppler ultrasound.

## Abbreviations

CPB: cardiopulmonary bypass
eGFR: estimated glomerular filtration rate
ICU: intensive care unit
IV: intravenous
IVC: inferior vena cava
LVEDD: end-diastolic dimension
LVEF: left ventricular ejection fraction
RI: resistive index
TAPSE: tricuspid annular plane systolic excursion
